# Comprehensive Phenotype of the p.Arg420his Allelic Form of Spinocerebellar Ataxia Type 13

**DOI:** 10.1007/s12311-013-0507-6

**Published:** 2013-08-03

**Authors:** SH Subramony, Joel Advincula, Susan Perlman, Raymond L. Rosales, Lillian V. Lee, Tetsuo Ashizawa, Michael F. Waters

**Affiliations:** 1 Department of Neurology, University of Florida College of Medicine, Box 100296, Gainesville, FL 32610 USA; 2 Department of Neuroscience, University of Florida College of Medicine, Box 100296, Gainesville, FL 32610 USA; 3 Western Visayas State University Medical Center, Iloilo City, Philippines; 4 Department of Neurology at the UCLA Medical Center, Los Angeles, CA USA; 5 Department of Neurology and Psychiatry, University of Santo Tomas, Manila, Philippines; 6 Child Neuroscience Center, Philippine Children’s Medical Center, Quezon City, Philippines; 7 McKnight Brain Institute, University of Florida, Gainesville, FL 32610 USA

**Keywords:** Spinocerebellar ataxia type 13, Ataxia, Voltage-gated potassium channel, Neurodegeneration, Neurogenetics

## Abstract

The p.Arg420His allelic form of spinocerebellar ataxia type 13 has been reported in a large Filipino kindred, as well as three European index cases, one with an affected offspring. Haplotype analysis has confirmed independent mutational events. All individuals share adult-onset, predominantly cerebellar signs and a slowly progressive course. However, a comprehensive phenotypic description has yet to be published on SCA13^p.Arg420His^. In this study, we present the results of a detailed neurological clinical and diagnostic testing on 21 mutation-positive members of a four-generation Filipino family to further define this disease, aiding diagnosis and prognosis.

**Electronic supplementary material:**

The online version of this article (doi:10.1007/s12311-013-0507-6) contains supplementary material, which is available to authorized users.

## Introduction

Spinocerebellar ataxia type 13 (SCA13) is an autosomal dominantly inherited disorder caused by mutations in *KCNC3* coding for the Kv3.3 voltage-gated potassium channel [1]. Of the three SCA13-causative mutations described, the p.Phe448Lys and p.Arg423His mutant alleles result in developmental phenotypes with delayed motor milestones, mental retardation, and epilepsy [2, 3] whereas the p.Arg420His mutation effects a neurodegenerative phenotype clinically resembling other SCA subtypes [1, 2]. In vitro experimental systems have shown that each mutation possesses distinct biophysical properties. The electrophysiological profiles of the p.Arg420His allele are consistent with a null mutation. Moreover, co-expression with a wild-type allele demonstrates a dominant negative effect that is specific to *Shaw* subtype voltage-gated potassium channels. In contrast, the p.Phe448Lys and p.Arg423His mutations alter the gating, conductance, and closing properties of the channel [1, 3].

There has been increasing identification of SCA subtypes in the last several years. Many of the common SCAs are related to unstable repeat expansion mutations though recently an increasing number of point mutations have been linked to similar phenotypes. The phenotypic overlap among different genotypes and phenotypic heterogeneity related to specific genotypes oftentimes prevents facile identification of the underlying mutation based on clinical features alone [4–6]. Common practice involves obtaining panels of diagnostic DNA tests covering all known ataxias at considerable expense. Accurate genotypic diagnosis brings closure to the evaluative process for patients and their families, permits improved genetic counseling, and facilitates participation in targeted research studies and clinical trials. Further, specific and well-characterized phenotypic features may aid the clinician in prioritizing the mutation analysis.

Here, we report detailed phenotypic features in a large Filipino kindred harboring the SCA13^p.Arg420His^ mutation, extend the limited clinical observations reported with the original linkage studies, and provide the most comprehensive description of this SCA13 allelic form.

## Methods

**Fig. 1:**
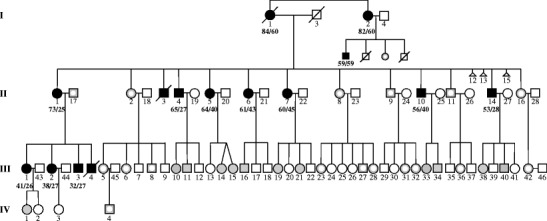
Pedigree showing an extended SCA13 Filipino family. *Filled black symbols* are clinically affected individuals with known c.1259G>A genotype. *Gray symbols* represent asymptomatic examined individuals with known genotype. *White insets* indicate two wild-type alleles. Of the remaining 12 at-risk individuals, nine are c.1259G>A heterozygotes and three are wild type. They are not identified for privacy concerns. *Bold numbers* indicate the ages at examination and disease onset

Forty-one members of a four-generation Filipino kindred segregating the *KCNC3* ^*p.Arg420His*^ allele were comprehensively examined, and blood samples were collected for mutation detection (Fig. 1). Data obtained included detailed medical and neurological histories: full neurological examination to obtain information on their oculomotor, bulbar, cerebellar, upper motor neuron, lower motor neuron, extrapyramidal and peripheral nervous systems, Montreal Cognitive Assessment (MoCA-P, Philippine version 7.1, 2010 administered by a trained local neuropsychologist), Scale for Assessment and Rating of Ataxia (SARA) score [7], “functional stage” indicating mobility status [8] (grade 0 being asymptomatic and grade 6 being bedbound and needing full help with activities of daily living), activities of daily living scale (Unified Huntington’s Disease Rating Scale (UHDRS IV)), and EQ-5D self-assessed quality of life scale. All data were obtained prior to mutation testing. Three affected members had nerve conduction studies performed. One underwent a 1-h sleep/awake EEG. MR imaging of the brain was obtained in five individuals, II-1, II-14, III-1, IV-1, and III-3. Serial scans for a mother–daughter pair (II-1/III-2) with a 5-year interval are included for longitudinal comparison. University of Florida Institutional Review Board approval for this study and signed informed consents for all patients have been retained. The original report of this family was limited to 11 affected individuals (I-1, II-1, II-4, II-5, II-6, II-7, II-10, II-14, III-2, III-3, and III-4) examined only for cerebellar, gait, and motor system abnormalities [2].

## Results

Of the 41 individuals studied, 21 (M/F 8:13) were found to harbor the p.Arg420His (c.1259G>A) mutation. Nine mutation-positive individuals (M/F 3:6) had not yet developed clinical symptoms. Mean age at onset among the 12 symptomatic individuals was 37.3 years (range 25 to 60) with a standard deviation (SD) of 12.7 years, and the mean age at examination was 54.7 ± 16 (SD) years (range 32 to 82). Mean duration of disease at examination was 17.6 ± 14.7 (SD) years (range 1 to 48). All symptomatic subjects demonstrated gait and limb ataxia. In two, cerebellar signs were limited to gait and lower limbs. Dysarthria was found in 10/12. As previously reported, none of the individuals had cerebellar oculomotor disturbance including nystagmus, abnormal pursuit, or saccadic dysmetria [9]. Ocular movements were full, saccadic speed was normal, and there was no ptosis. Three patients, none with a weak cough, reported dysphagia. Decreased vibration sense involving toes occurred in 6/12 (age at examination was 53 to 82 years with diabetes in two) though no additional sensory abnormalities were observed. Brisk deep tendon reflexes without spasticity were found in five patients, being confined to the lower limbs in two and to the upper limbs in two. One individual (diabetic) had absent deep tendon reflexes. None had extensor plantar reflex, chorea, dystonia, tremor, or other extrapyramidal signs. Three individuals reported mild bladder control issues, and one had nocturnal enuresis. Two persons reported occasional sudden trunk movements consistent with myoclonic jerks though no myoclonus was observed during examination. None reported histories of seizures or episodic ataxia (Table 1).

**Table 1:** Summary of pertinent clinical features among 12 symptomatic subjects with SCA13^R420H^

Clinical characteristic	Total
Age at onset	37.25 ± 12.7 (range 25–45) years
Male/female (complete pedigree)	10:14
Dysarthria	10/12 (83.3)
Dysphagia	3/12 (25)
Gait ataxia	12/12 (100)
Lower limb ataxia	12/12 (100)
Upper limb ataxia	10/12 (83.3)
Atrophy/weakness	0/12 (0)
Spasticity	1/12 (8.3)
Brisk deep tendon reflexes	5/12 (41.7)
Electrophysiological signs	0/12 (0)
Vibration sensory loss (toes)*	6/12 (50)
Bladder dysfunction	3/12 (25)
Epilepsy/seizures	0/12 (0)
History of myoclonic jerks	2/12 (16.7)
Episodic ataxia features	0/12 (0)
Impaired cognition (MoCA-P)**	14/21 (67)

Numbers within parenthesis are percentages

*Age at exam 53-82 years; 2 with diabetes

**All mutation+ individuals included. See text for details

The mean SARA score at examination was 12.8 ± 8.7 (SD) (range 1 to 32.5), and the mean functional stage was 2.9 ± 1 (SD) (range 2–5). The annualized SARA score increase, as a function of disease duration, was 0.73 per year. Three persons used canes, one at the onset of disease at age 60 and the others after 13 and 20 years of disease onset. One person started using a walker after 28 years of disease progression. A 73-year-old woman with a 48-year disease duration was wheelchair-bound. The remaining seven patients continued to ambulate unassisted, including three subjects with a disease duration of 10 to 25 years. The mean score on the EQ-5D visual analog scale was 65.5 ± 13.6 (SD) (100 being the best possible and 0 being the worst possible self-perceived health), and the mean UHDRS IV functional score was 18.5 ± 4.5 (SD) (25 being the best and 0 being the worst functional capacity).

The MoCA-P revealed 14 of 21 (67 %) scores in the abnormal range, which were less than 26 (23 ± 3.9 SD, range 15–29). The most common deficit was delayed recall in ten subjects and abnormal verbal fluency in eight. Visuospatial skills (clock or cube drawing) were also impaired in eight. However, these results must be interpreted with caution as (1) there was little correlation between MoCA-P severity and disease duration (Pearson *r* = −0.38) or SARA score (Pearson *r* = −0.37), (2) seven (33 %) symptomatically affected individuals scored in a normal range, and (3) 6 of 18 unaffected noncarriers (33 %) who were tested also scored less than 26 sharing delayed recall as the most common deficit (26 ± 2.3 SD, range 21–29).

**Fig. 2:**
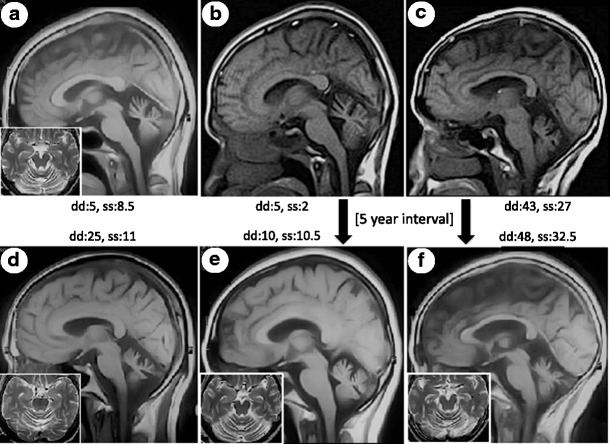
MR T1 midline sagittal and T2 axial sequences (*inset*) suggest progressive cerebellar atrophy with disease duration (dd) and SARA severity (ss) (**a** disease duration 5 years, SARA 8.5; **d** disease duration 25 years, SARA 11). Panels **b** and **e** (daughter) and **c** and **f** (mother) are repeated studies with a 5-year interval between initial and subsequent examination and imaging (**a**, **b** 33/38 years; **c**, **d** 68/73 years). There is little change in the degree of cerebellar vermian atrophy over 5 years though progression of disease was evident in an increased SARA from 2 to 10.5 (**b**, **e**) and from 27 to 32.5 (**c**, **f**). The brain stem and pons remain intact in spite of a 48-year disease duration (**f**)

MR scans of the brain in four test subjects revealed isolated cerebellar atrophy (Fig. 2). In two individuals in which serial scans with a 5-year interval was available, no significant progression of atrophy could be appreciated in spite of clear progression of SARA disease severity (Fig. 2b, e, c, f). Sensory and motor nerve conduction studies were performed on three symptomatic individuals and revealed no pathological findings to indicate a peripheral neuropathy. One-hour sleep/awake EEG in a symptomatic individual (SARA 10.5) was normal without evidence for epileptic discharges, focality, slowing, or other pathologic patterns.

The mean age at examination of the nine mutation-positive asymptomatic subjects was 24 ± 7.3 (SD) years. Among these subjects, four had brisk deep tendon reflexes, one of whom also had mild lower extremity spasticity and a slight tremor. A fifth patient demonstrated a minimally abnormal heel to shin test. In all other respects, their neurological examination was normal. Seven of the nine mutation-positive asymptomatic subjects had a SARA score of 0 and two obtained scores of 1 and 1.5 but self-described as asymptomatic. Mean EQ-5D VAS score was 80.1 ± 16.4 (SD), and the UHDRS score was 23.5 ± 2.5 (SD).

## Discussion

An adult-onset, slowly progressive ataxic syndrome characterizes the phenotype of SCA13 related to the *KCNC3* ^p.Arg420His^ mutation with a widely ranging age at onset. The clinical signs were primarily purely cerebellar, though no oculomotor pathologies related to cerebellar degeneration were detected. Brisk deep tendon reflexes were found in less than half of the subjects without associated marked spasticity or upper motor neuron signs suggestive of upper motor neuron dysfunction. Half of the subjects had diminished vibration sense in the toes though two of them were diabetic and the others were over 50 years of age, confounding the determination of mutation causality. No evidence for extrapyramidal or anterior horn cell involvement was found. Nerve conduction studies performed on three subjects were entirely normal with no evidence for peripheral neuropathy. None of the individuals had seizures or episodic symptoms.

Though limited descriptions of SCA13^p.Arg420His^ have been published previously, this report provides an in-depth phenotypic characterization of the largest sample group to date including cognitive assessment, formal ataxia scale ratings, functional staging, and self-assessed quality of life indicators in presymptomatic, early, middle-, and late-stage patients. This report is limited in that it describes these features in a single kindred, though strengthened in that it includes two pedigree branches and four generations of subjects. Moreover, it provides an important foundation on which the natural history of SCA13^p.Arg420His^ may be characterized as it includes examination of presymptomatic patients as well as those with SARA severity scales ranging from 1 to 32.5, functional staging of 0.5 to 5, and disease duration of 1 to 48 years.

Other reports of this SCA13 allele include four individuals from three different lineages, all discovered by large-scale DNA sequencing from unknown ataxia repositories [3]. One individual was described similarly to the current cohort with a disease duration of approximately 20 years at age 43, cerebellar signs, ataxic gait, and clear progression of symptoms. A small kindred, one aged 66 years with onset at 42 years, and their offspring with a 3-year duration at age 31 years also shared cerebellar signs, gait ataxia, and disease progression but were also observed to have pyramidal signs and, in the younger subject, a seizure disorder [10]. This observation potentially extends the phenotype to include epilepsy, which has been described in the p.Arg423His allelic form of SCA13 [11]. The final patient, aged 57 years with 6 years of symptoms, shared cerebellar and pyramidal signs and gait ataxia. No follow-up was available to assess for disease progression. Though the number of affected symptomatic individuals in the pedigree is too small to perform definitive studies on genetic modifiers of SCA13^p.Arg420His^, the number of at-risk individuals in this family, as well as the potential discovery of additional pedigrees, may provide the opportunity for this type of analysis in the future.

SCA13^p.Arg420His^ is slowly progressive. The annual increase in SARA score based on this cross-sectional examination was much slower than for other SCAs associated with extracerebellar signs such as SCAs 1, 2, and 3 [12]. Only three individuals used a cane, and only one was wheelchair-bound despite mean disease duration of 17.6 years. The minimal decrease in the mean UHDRS IV functional score reinforces the slow progression.

Contrary to the initial impression that this mutation is not associated with cognitive impairment, we found evidence for mild abnormalities in a number of affected individuals. Seven of 12 subjects scored in the abnormal range with deficits in delayed recall, verbal fluency, and visuospatial tasks.

The mutation-positive but asymptomatic subjects generally had normal examinations excepting brisk tendon reflexes in four. However, a slight tremor, mild heel-to-shin ataxia, and minimal spastic catches were also observed. Additionally, imaging evidence suggests that cerebellar atrophy may significantly precede overt clinical symptomatology. Thus, though referencing SCA13^p.Arg420His^ as an adult-onset, slowly progressive neurodegenerative phenotype is clinically accurate, it may underestimate the actual onset of disease pathology. The evolution of ataxic features and when these subjects become frankly symptomatic remains to be determined.

## Summary

An autosomal dominant ataxia with isolated cerebellar signs, slow progression, mild cognitive impairment, and absence of cerebellar oculomotor signs may suggest the p.Arg420His allelic form of SCA13. Isolated cerebellar atrophy on MRI with little change over many years would further strengthen this conclusion.

## Electronic Supplementary Material

Below is the link to the electronic supplementary material.

Supplemental Figure MR T1 weighted midline sagittal image illustrating normal cerebellar volume occupying the posterior fossa. (PPTX 366 kb)
