# Supplementary material for: Comprehensive Phenotype of the p.Arg420his Allelic Form of Spinocerebellar Ataxia Type 13
Source: Cerebellum. 2013 Aug 3;12(6):932–6. doi: 10.1007/s12311-013-0507-6 (PMC3824261; doi:10.1007/s12311-013-0507-6)
Supplement: Supplementary file 1 — MR T1 weighted midline sagittal image illustrating normal cerebellar volume occupying the posterior fossa. (PPTX 366 kb) [file 12311_2013_507_MOESM1_ESM.pptx]

## Slide 1
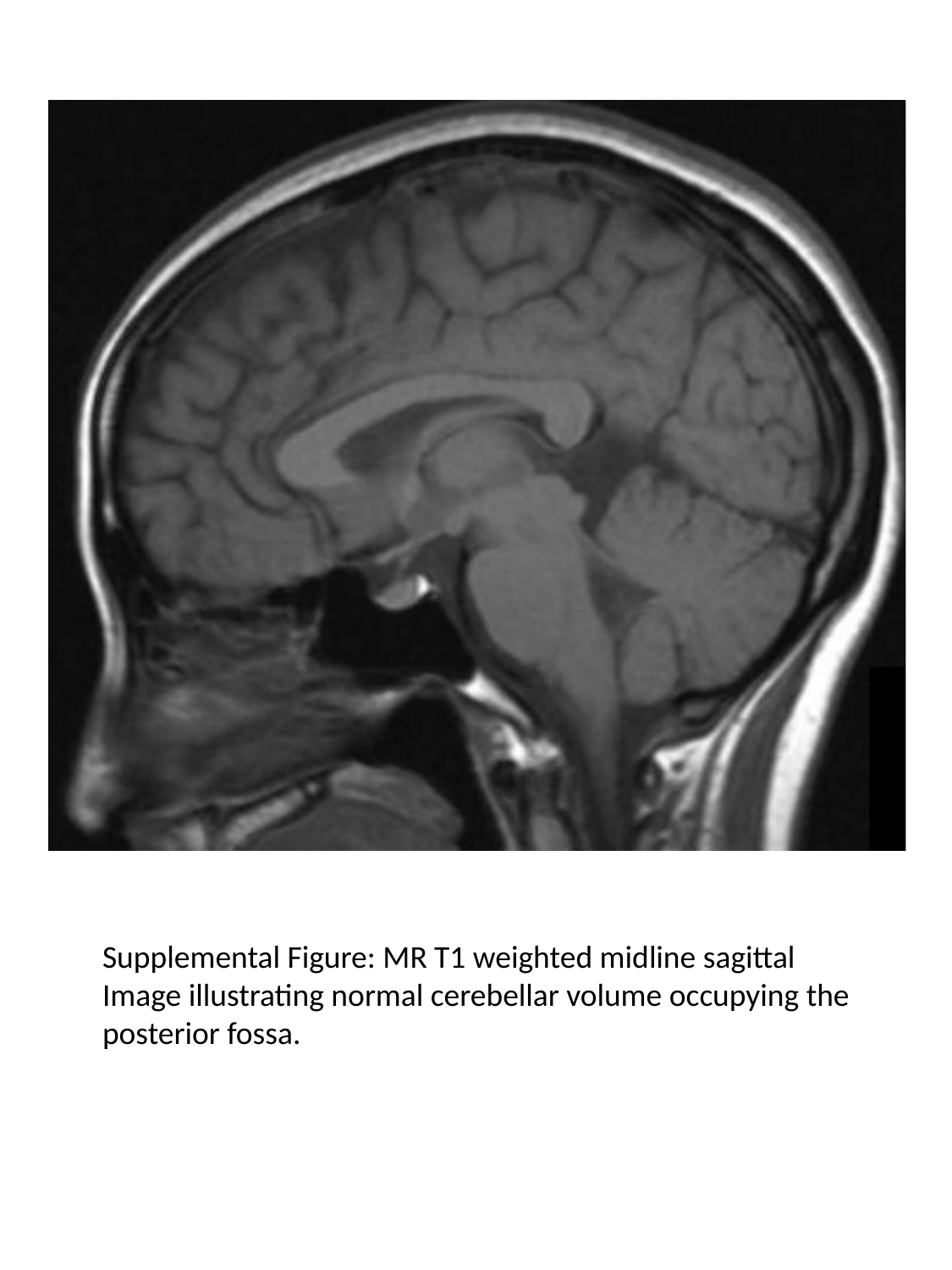

Supplemental Figure: MR T1 weighted midline sagittal
Image illustrating normal cerebellar volume occupying the
posterior fossa.
